# *Mrassf1a*-Pap, a Novel Methylation-Based Assay for the Detection of Cell-Free Fetal DNA in Maternal Plasma

**DOI:** 10.1371/journal.pone.0084051

**Published:** 2013-12-31

**Authors:** Jessica M. E. van den Oever, Sahila Balkassmi, Tim Segboer, E. Joanne Verweij, Pieter A. van der Velden, Dick Oepkes, Egbert Bakker, Elles M. J. Boon

**Affiliations:** 1 Department of Clinical Genetics, Laboratory for Diagnostic Genome Analysis (LDGA), Leiden University Medical Center, Leiden, The Netherlands; 2 Department of Obstetrics, Leiden University Medical Center, Leiden, The Netherlands; 3 Department of Ophthalmology, Leiden University Medical Center, Leiden, The Netherlands; Tel Aviv University, Israel

## Abstract

**Objectives:**

*RASSF1A* has been described to be differentially methylated between fetal and maternal DNA and can therefore be used as a universal sex-independent marker to confirm the presence of fetal sequences in maternal plasma. However, this requires highly sensitive methods. We have previously shown that Pyrophosphorolysis-activated Polymerization (PAP) is a highly sensitive technique that can be used in noninvasive prenatal diagnosis. In this study, we have used PAP in combination with bisulfite conversion to develop a new universal methylation-based assay for the detection of fetal methylated *RASSF1A* sequences in maternal plasma.

**Methods:**

Bisulfite sequencing was performed on maternal genomic (g)DNA and fetal gDNA from chorionic villi to determine differentially methylated regions in the *RASSF1A* gene using bisulfite specific PCR primers. Methylation specific primers for PAP were designed for the detection of fetal methylated *RASSF1A* sequences after bisulfite conversion and validated.

**Results:**

Serial dilutions of fetal gDNA in a background of maternal gDNA show a relative percentage of ∼3% can be detected using this assay. Furthermore, fetal methylated *RASSF1A* sequences were detected both retrospectively as well as prospectively in all maternal plasma samples tested (n = 71). No methylated *RASSF1A* specific bands were observed in corresponding maternal gDNA. Specificity was further determined by testing anonymized plasma from non-pregnant females (n = 24) and males (n = 21). Also, no methylated *RASSF1A* sequences were detected here, showing this assay is very specific for methylated fetal DNA. Combining all samples and controls, we obtain an overall sensitivity and specificity of 100% (95% CI 98.4%–100%).

**Conclusions:**

Our data demonstrate that using a combination of bisulfite conversion and PAP fetal methylated *RASSF1A* sequences can be detected with extreme sensitivity in a universal and sex-independent manner. Therefore, this assay could be of great value as an addition to current techniques used in noninvasive prenatal diagnostics.

## Introduction

Over the past years, the use of cell-free fetal DNA (cffDNA) for noninvasive prenatal diagnosis (NIPD) has proven its clinical potential in a wide range of fields. Although the possibilities for using cffDNA in NIPD are numerous, they do require highly sensitive and specific techniques to detect the low levels of fetal sequences in the pool of maternal plasma DNA early in gestation.

For the detection and/or quantification of fetal DNA, many investigators have based their strategy on the detection of Y-chromosomal-specific sequences (*SRY* and *DYS14*), or on the use of paternally inherited SNPs or polymorphic loci that are either absent or different in the mother [1]–[5]. Even though Y-chromosomal sequences can be detected using several different techniques with high sensitivity and specificity early in gestation, a positive result can only be obtained in pregnancies with a male fetus. Additional detection of paternally inherited sequences could be used to discriminate between a true negative result in case of a female pregnancy, or a false negative result in case of low levels of circulating cffDNA. However, these methods are quite laborious since both biological parents need to be tested along with the plasma sample and not all SNPs and loci tested will be informative. Therefore, a large panel of different markers need to be tested for each individual case [5].

Other fetal identifiers have been described which are based on epigenetic differences between fetus and mother. These differences are caused by so-called genomic imprinting and are characterized by differential expression of maternally and paternally inherited genes due to transcriptional silencing of either one of these genes through DNA methylation [6]. The use of genomic imprinting in NIPD was first shown by the group of Poon *et al*. displaying differences in methylation status between fetal and maternal sequences in a region of the human *IGF2-H19* locus [7]. Since it has been shown that cffDNA in maternal plasma originates from trophoblast cells of the placenta, the search for differentially methylated patterns has focused on genes expressed in placental tissues [8]–[18]. One of such genes that have been reported to be differentially methylated between mother (hypomethylated) and fetus (hypermethylated) is Ras-Association Domain Family Member 1, transcript variant A (*RASSF1A*) [13], [18]–[24]. Previous studies used these differences in methylation in *RASSF1A* to confirm the presence of cffDNA in maternal plasma, independent of fetal sex and without the restriction of only detecting paternally inherited sequences [13], [18]–[24]. Methylation-sensitive restriction enzyme digestion, (Real-Time) methylation specific PCR (MSP), mass spectrometry and bisulfite conversion in combination with direct sequencing were the main techniques used in these studies. Some of the aforementioned techniques require a relatively high DNA input. This may indicate that not all of these techniques are sensitive enough to detect the low levels of cffDNA in maternal plasma early in gestation. We have previously shown that Pyrophosphorolysis-activated polymerization (PAP) is a highly sensitive method for the detection of fetal sequences in a large pool of maternal plasma [25], [26]. PAP was initially developed to detect rare known mutations with high selectivity in an excess of wild-type template [27]. It utilizes unidirectional (PAP) or bidirectional (bi-PAP) blocked oligonucleotides on the 3′end. These blocks need to be removed by pyrophosphorolysis for DNA extension to occur. This is only possible when the oligonucleotides completely match the template strand. This makes PAP a highly specific and sensitive method to use in NIPD [25]–[29].

In this study we have used this method to develop a new universal sex-independent methylation-based assay to detect fetal methylated *RASSF1A* (*mRASSF1A*) sequences in maternal plasma for NIPD.

## Materials and Methods

### Samples

Written informed consent was obtained and this study was approved by the Medical Ethics Committee (CME) of the Leiden University Medical Center. Maternal peripheral blood samples (10–20 mL) were collected in EDTA coated tubes from pregnant women for noninvasive fetal sexing at the Laboratory for Diagnostic Genome Analysis of the Leiden University Medical Center (LUMC), Leiden, the Netherlands. Maternal blood samples (n = 71) were drawn at a median gestational age of 10.6 weeks (range 8.0–18.1 wks.) and were processed within 24 hrs. after collection as described previously [30]. The retrospective samples used were previously tested for fetal sexing (n = 60) using a combination of Real-Time PCR and Pyrophosphorolysis-activated polymerization (Y-PAP) for the detection of Y-chromosomal sequences as previously described [25]. All fetal gender was confirmed by karyotyping or after birth. In the prospective samples (n = 11) fetal sexing was determined using a combination of tests mentioned above, supplemented with Real-Time PCR detection of a panel of 8 high frequency paternal deletion/insertion polymorphisms [2]. As a control, anonymized plasma control samples from males (n = 21) and non-pregnant females (age>48, n = 24) were used.

### DNA isolation

Cell-free DNA was isolated from plasma with the EZ1 Virus Mini Kit v2.0 on the EZ1 Advanced (QIAGEN, Venlo, The Netherlands; www.qiagen.com) according to the manufacturer's instructions with an input volume of 800 (2*400) µL plasma and an elution volume of 120 (2*60) µL.

### Bisulfite conversion

Bisulfite conversion was performed using the EZ DNA Methylation-Gold™ kit (Zymo Research, USA) according to manufactures' instructions, with an input of 100 ng gDNA per reaction (maximum DNA reaction volume of 50 µL) and an elution volume of 10 µL. Bisulfite conversion of plasma DNA was performed as mentioned previously, with an input of 2*50 µL total cell-free DNA (cfDNA) from plasma per bisulfite reaction. (N.B. two corresponding plasma DNA samples were pooled after conversion and purified over 1 column). Elution volume used was 10 µL.

### Bisulfite sequencing and *mRASSF1A*-PAP primer design

**Figure 1:**
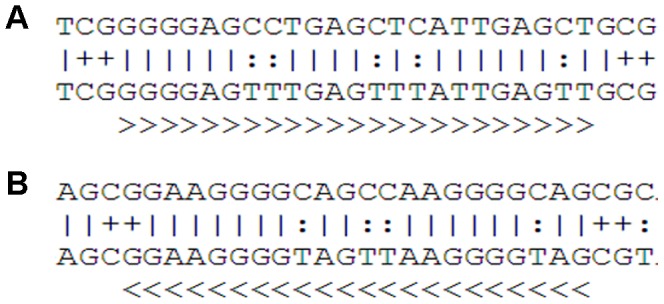
Sequences after Methprimer prediction. Predicted sequences of the *RASSF1A* for Bisulfite Specific Primers (BSP) design using Methprimer [31]. BSP primers are located outside differentially methylated regions. Methylated nucleotides are indicated with +, unmethylated nucleotides with: and other nucleotides with |. A: The predicted sequence of the BisB forward primer (indicated as >>>). B: The predicted sequence of the BisB reverse primer (indicated as <<<).

Two sets of Bisulfite Sequencing Primers (BSP) containing an M13 tag for Sanger sequencing were designed for two subsequent fragments (BisA 191 bp and BisB 297 bp, Fig. 1, Table 1) in the promoter region of the *RASSF1A* gene (NM_007182.4) outside predicted CpG islands or other potentially methylated cytosines using MethPrimer v1.1 beta [13], [31], [32]. After bisulfite conversion, we assessed methylation patterns of these two regions by conventional Sanger sequencing using these 2 sets of BSP-M13 primers and SeqScape Software (Applied Biosystems). Three sets of fetal gDNA derived from chorionic villus samples (CVS) and corresponding maternal gDNA sequences from maternal blood cells were compared to determine differentially methylated regions of the *RASSF1A* gene at nucleotide level. Methylation specific PAP primers for the detection of *mRASSF1A* were subsequently designed and a so-called bi-PAP reaction was performed.

**Table 1:** Bisulfite sequencing primers and PAP primers.

Target	Name	Sequence (5′ – 3′)	Product size (bp)	Primer type
*RASSF1A*	RASSF1A_BISaF-**M13**	**TGTAAAACGACGGCCAGT** AGTTTTTCTATTTACCTTTTTATTG	227*	BSP
*RASSF1A*	RASSF1A_BISaR-**M13**	**CAGGAAACAGCTATGACC** AACTCAATAAACTCAAACTCCCC		BSP
*RASSF1A*	RASSF1A_BISbF-**M13**	**TGTAAAACGACGGCCAGT** GGGGAGTTTGAGTTTATTGAGTT	333*	BSP
*RASSF1A*	RASSF1A_BISbR-**M13**	**CAGGAAACAGCTATGACC** CTACCCCTTAACTACCCCTTCC		BSP
*RASSF1A*	M-RASSF1A_PAPF2	GTTGGAGCGTGTTAACGCGTTGCGTAT-ddC	110	PAP
*RASSF1A*	M-RASSF1A_PAPR2	ACGTAACGAACCCCGCGAACTAAAAACGATAA-ddC		PAP

Primer sequences. M13 tag used for Sanger sequencing is depicted in bold. BSP: Bisulfite Specific Primer, PAP: Pyrophosphorolysis-activated Polymerization.

*Product sizes for BSP primers are including the M13 tags.

### mRASSF1A-PAP

The *mRASSF1A*-PAP reaction mixture contained 1x PAP-PCR buffer (250 mM Tris-HCl pH 7.5 (Gibco, Life Technologies Corporation), 80 mM (NH_4_)_2_SO_4_ (J.T. Baker), 17.5 mM MgCl_2_ (J.T. Baker), 125 µM of each of the four dNTP's (Thermo Scientific), 450 µM Na_4_PPi pH 8.0 (Sigma-Aldrich)), 2.5 IU Klentaq S (ScienTech Corp), 4 µM of each PAP-primer (Biolegio, Nijmegen, the Netherlands, Table 1) and 10 µL of bisulfite converted cfDNA from maternal plasma. Cycling conditions were 15 s 94°C, 40 s 60°C, 40 s 64°C, 40 s 68°C and 40 s 72°C for a total of 45 cycles. PAP reaction product was visualized on a 3.5% 1x TBE agarose gel.

As an internal negative control, maternal gDNA from the buffy coat (input 100 ng) was always converted and analyzed together with the cfDNA isolated from the corresponding maternal plasma sample. A fully methylated human cell line (CpGenome, S7821, Merck Millipore) and/or a gDNA sample from CVS (both 100 ng input per reaction) were used as positive controls to check the bisulfite conversion and the PAP reaction. For the latter, this control had been converted in an independent separate reaction, aliquoted and stored at −20°C until further use.

Serial dilutions (range 1000–7 pg) of fetal gDNA from CVS in H_2_O were performed to determine the analytical sensitivity of the assay. In addition, comparable serial dilutions of fetal gDNA in a background of 1000 pg maternal gDNA were performed. Input mentioned is the total amount of fetal gDNA per bisulfite conversion reaction.

## Results

### Determination of differentially methylated regions in *RASSF1A*

**Figure 2:**
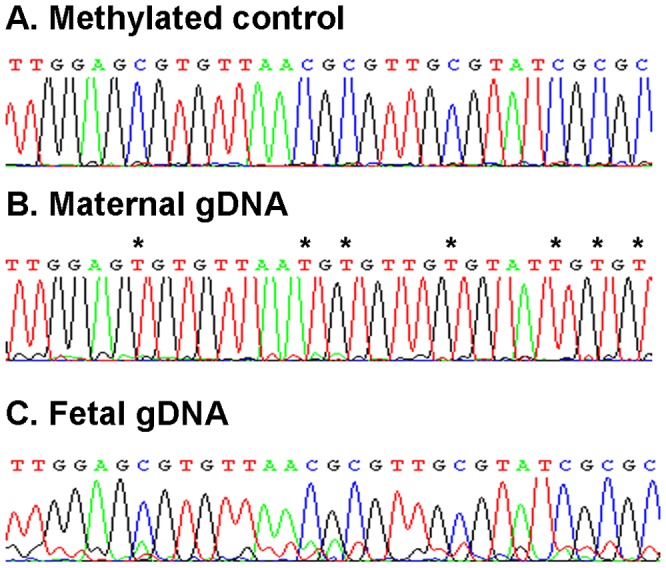
Differentially methylated regions after bisulfite sequencing. Sanger sequencing results for *RASSF1A* of a fully methylated control cell line (A), maternal gDNA (B) and fetal gDNA derived from CVS (C) after bisulfite sequencing. A representative part of the complete sequence is shown. All unmethylated cytosines are converted to uracil after bisulfite sequencing. Differences between maternal and fetal (methylated) sequences are indicated with an *.

**Figure 3:**
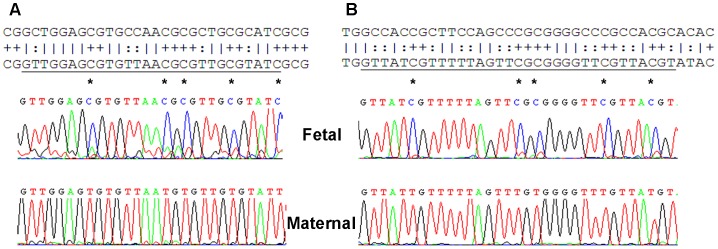
Predicted and confirmed sequences for PAP-primer design. Sequences of the *RASSF1A* gene were analyzed after bisulfite sequencing of maternal gDNA and fetal gDNA derived from CVS. Differentially methylated regions of the BisB region predicted by MethPrimer [31] could be confirmed using bisulfite sequencing. Both forward (A, upper panel, underlined) and reverse PAP-primer (B, upper panel, underlined) are specific for fetal sequences (middle panels) after bisulfite conversion and both primers have several mismatches to the maternal sequences (lower panels). Mismatches between fetal specific PAP-primers and maternal sequences are indicated with an * for each primer.

To determine regions in the *RASSF1A* gene which are differentially methylated between mother and fetus, bisulfite sequencing was performed on maternal gDNA and fetal gDNA from CVS (n = 3 sets). Two different regions (BisA and BisB) were analyzed by conventional Sanger sequencing using two sets of BSP-M13 primers (Fig. 1, Table 1). Differentially methylated sequences were found in both regions (Fig. 2). *mRASSF1A*-PAP primers PAP primers were designed in the region covered by the BisB BSP primers and are specific for fetal methylated sequences after bisulfite conversion (Figure 3, Table 1). This region was also previously described by the group of Chiu and colleagues [13]. We considered this region the most suitable for PAP primer design since it contains many methylated cytosines in the fetal (hypermethylated) sequences, while in the mother, these cytosines are unmethylated and will convert into uracil after bisulfite conversion. This resulted in 5 mismatches between each PAP primer and maternal DNA template and will increase the specificity of this assay (Fig. 3). To increase specificity of the PAP primers even more, the length of the oligonucleotides was at least 28 nt. In addition, this assay was designed as a bi-PAP, containing a 3′ddC block on both the forward as well as the reverse primer.

### Analytical sensitivity and specificity of the *mRASSF1A*-PAP assay

**Figure 4:**
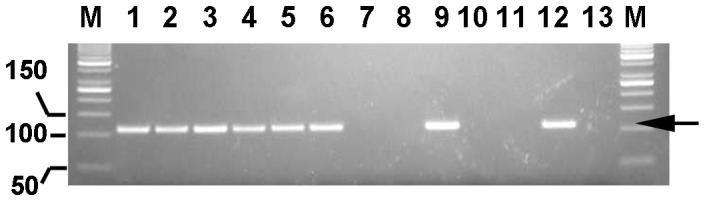
*mRASSF1A*-PAP serial dilution range of gDNA from CVS. Serial dilutions were performed with gDNA from CVS in a background of 1000(pg) mentioned is the total amount of fetal gDNA. M = 50 bp marker, 1 = 1000 pg, 2 = 500 pg, 3 = 250 pg, 4 = 125 pg, 5 = 60 pg, 6 = 30 pg, 7 = 15 pg, 8 = 7 pg, 9 =  positive control for bisulfite conversion, 10 = NTC for bisulfite conversion, 11 =  negative control for bisulfite conversion (non-bisulfite converted fetal gDNA), 12 =  positive control for *mRASSF1A*-PAP, 13 = NTC for *mRASSF1A*-PAP. A 110 bp product (arrow) is obtained in cases where *mRASSF1A* sequences could be detected using *mRASSF1A*-PAP.

The analytical sensitivity of the *mRASSF1A*-PAP assay was first determined by testing serial dilutions of gDNA derived from CVS in water. Our results show that this assay is sensitive enough to detect fetal sequences in amounts as low as 16 pg in a 50 µL sample reaction volume (data not shown). To simulate the situation in maternal plasma, gDNA from CVS was serially diluted in a background of maternal gDNA. Our data show that in a background of 1000 pg maternal gDNA, as low as 30 pg of fetal gDNA can be detected, representing a relative percentage of around 3% (Fig. 4). These serial dilutions thus showed that this assay is highly sensitive.

To demonstrate that this assay is also highly specific for methylated fetal DNA sequences, several controls were tested. As an internal negative control, corresponding maternal gDNA samples were always converted and analyzed in parallel to the maternal plasma samples. No *mRASSF1A* specific bands were observed in these samples. In addition, anonymized plasma samples were tested from non-pregnant females (age >48, n = 24) and males (n = 21). Also, no *mRASSF1A*-specific products were observed here. Therefore, the assay is also very specific since no false positives were present in all control samples tested (n = 116), resulting in an analytical sensitivity and specificity of 100% (95% CI 97.4%–100%).

### Testing of retrospective and prospective clinical samples

In a retrospective study, fifty three samples previously tested for fetal gender using a combination of Real Time PCR and Y-PAP [25] and indicated as undetermined (no Y chromosomal sequences detected), were tested for the presence of cffDNA using the *mRASSF1A*-PAP assay (Table 2). Fetal *mRASSF1A* sequences were detected in all maternal plasma samples tested (n = 53). As a control, the presence of *mRASSF1A* was also confirmed in 7 samples already tested positive for Y-chromosomal sequences. In these samples the presence of fetal DNA could be confirmed both in a sex-dependent and sex-independent assay, showing that the results from the *mRASSF1A* detection are concordant with the detection of Y chromosomal sequences. The data also confirmed that since this assay is sex-independent, it can be applied to all pregnancies. Altogether, the presence of cffDNA in maternal plasma was shown retrospectively with a sensitivity and specificity of 100% (95% CI 95.0%–100%).

**Table 2:** Summary of sample characteristics from the retrospective study.

Samples	Gestational Age (range in weeks)	*SRY* Real-Time PCR	Y-PAP	*mRASSF1A*	Fetal gender	Confirmation (karyo/birth)	Concordance
n = 53	8.0–18.1	Undet.	Undet.	Pos.	Female	Yes	Yes
n = 7	9.0–15.4	Y	Y	Pos.	Male	Yes	Yes

Summary of sample characteristics used in the retrospective study. Undet.: Undetermined (e.g. no Y chromosomal sequences were detected); Pos: positive; Y: Y chromosomal sequences were detected; Y-PAP: Y-chromosomal specific PAP-assay; Karyo: Full karyotyping performed on these samples; birth; fetal gender confirmed at birth.

Moreover, in a prospective study, several clinically relevant samples (n = 11) were tested with the *mRASSF1A*-PAP assay in parallel to our current diagnostic protocol for fetal sexing using the detection of Y-chromosomal sequences and, in case of a negative (e.g. undetermined or no Y chromosomal sequences detected) result, additional testing for 8 paternal deletion/insertion polymorphisms (Table 3) [2], [25]. Although a panel of high frequency polymorphisms was used, informative polymorphisms were either not present (n = 4), not inherited (n = 3) or results for the detection of these polymorphisms did not meet our quality criteria (e.g. at least 2/3 Ct values ≤40) used for diagnostics (n = 1) (Table 3). In parallel, these samples were tested using the *mRASSF1A*-PAP. In all 11 cases tested, the presence of fetal sequences could be confirmed using this new assay. In combination with both Real-Time PCR and Y-PAP results fetal gender could be determined as female (Table 3) and in all cases, our results were concordant with fetal gender determined after additional testing later on in gestation or after birth (Table 3).

**Table 3:** Sample characteristics from the prospective study.

Sample	Gestational Age (weeks)	*SRY* Real-Time PCR	Y-PAP	# IF Pols	# IF Pols detected	*mRASSF1A*	Fetal gender	Confirmation (karyo, QF-PCR, US, birth)	Concor-dance
1	10.7	Undet.	Undet.	3 IF	0 IF^a^	Pos.	Female	QF-PCR	Yes
2	8.6	Undet.	Undet.	1 IF	0 IF^a^	Pos.	Female	QF-PCR	Yes
3	13.6	Undet.	Undet.	0^b^ IF	-	Pos.	Female	US	Yes
4	9.0	Undet.	Undet.	0^b^ IF	-	Pos.	Female	US	Yes
5	10.4	Undet.	Undet.	1 IF	1 IF	Pos.	Female	Karyotyping	Yes
6	8.1	Undet.	Undet.	1 IF	1 IF	Pos.	Female	QF-PCR	Yes
7	9.0	Undet.	Undet.	1 IF	0 IF^c^	Pos.	Female	US	Yes
8	14.0	Undet.	Undet.	0^b^ IF	-	Pos.	Female	Birth	Yes
9	8.3	Undet.	Undet.	2 IF	0 IF^a^	Pos.	Female	US	Yes
10	9.0	Undet.	Undet.	1 IF	1 IF	Pos.	Female	QF-PCR	Yes
11	8.7	Undet.	Undet.	0^b^ IF	-	Pos.	Female	US	Yes

Sample characteristics of clinical samples (prospective study). Undet.: Undetermined (e.g. no Y chromosomal sequences were detected); IF: Informative; Pols: Polymorphisms; Pos: positive; QF PCR: Quantitative Fluorescent PCR; US: Ultrasound; birth; fetal gender confirmed at birth.

^a^ No informative polymorphisms detected/inherited,

^b^ no informative polymorphisms present,

^c^ results did not meet our quality criteria used in diagnostics (only 1/3 Ct values ≤40).

## Discussion

We have developed a novel sex- and polymorphism independent, methylation-based diagnostic test for the detection and confirmation of fetal DNA sequences in maternal plasma. In contrast to methods most widely used in noninvasive diagnostics (e.g. detection of Y chromosomal sequences or paternal polymorphisms), this assay can be applied to all pregnancies. Our test, based on the epigenetic differences between maternal (hypomethylated) and fetal (hypermethylated) *RASSF1A* sequences, was found to be 100% reliable.

Differentially methylated regions between mother and fetus have previously been identified in several genes. However, this was mainly done by techniques such as cloning, mass spectrometry and array. These techniques only produced methylation patterns without high resolution since CpG islands were analyzed as a whole [7], [12], [17]–[19], [21]. As we were interested in designing sequence-specific primers, we needed to study these methylation patterns at nucleotide level and therefore decided to perform bisulfite sequencing. The sequences of the BSP primers are located around predicted CpGs and other possible methylated cytosines [31]. Since these sequences are not influenced by bisulfite conversion, it is possible to study differentially methylated regions, both before and after conversion and subsequently to design methylation specific primers for Pyrophosphorolysis-activated Polymerization (PAP).

PAP was initially developed to enhance the specificity of allele-specific PCR for detection of known mutation in the presence of an excess of wild-type allele [27]–[29]. PAP requires an allele specific oligo with a dideoxyoligonucleotide block at the 3′end. If and only when the sequence of the oligo completely matches the template strand, the dideoxyoligonucleotide can be removed in the presence of pyrophosphate before the oligo can be extended subsequently. We have designed the *mRASSF1A*-PAP primers specific to the fetal (hypermethylated) sequences after bisulfite conversion. Compared to these fetal sequences, maternal (hypomethylated) sequences will differ quite extensively after bisulfite conversion, resulting in several mismatches between each primer and the maternal DNA template. This will prevent the PAP reaction from occuring since the 3′ block cannot be removed prior to extension, which makes this assay very specific. Although many other methods for minority allele enrichment have been described, PAP has been described to provide the highest selectivity [33]. This selectivity could even be enhanced using a bidirectional modification of two opposing allele-specific 3′ dideoxyoligonucleotides [27]–[29], [33]. The *mRASSF1A*-PAP is based on this bi-directional principle. We previously demonstrated the use of PAP for noninvasive fetal sex determination using a combination of Real-Time PCR and PAP for the detection of Y-chromosomal sequences [25]. This was successfully validated in our facility by testing a large amount of samples for noninvasive fetal sexing (n = 213), resulting in a diagnostic sensitivity and specificity of both 100% (95% CI 98.6%–100%) (unpublished data). Samples from the latter validation study were used for this *mRASSF1A*-PAP validation study as well. In daily clinical practice, we have also tested the *mRASSF1A*-PAP by using this assay in parallel with routine diagnostics for noninvasive fetal sexing. For the cases with undetermined results (e.g. no Y chromosomal sequences detected [25] we started out testing the *mRASSF1A*-PAP in parallel with Real-Time PCR detection of a panel of 8 high frequency paternal polymorphisms [2]. In some cases, no informative polymorphisms were present that could be used for a diagnostic conclusion. Thus despite using a panel of polymorphisms, the presence of cffDNA in maternal plasma could not be confirmed in 67% of the cases. *mRASSF1A*-PAP was also performed on these samples. In these cases *mRASSF1A*-PAP results were positive and fetal gender was determined and indeed confirmed as female showing that this assay could serve as a valuable supplemental test in diagnostics.

However, there are exceptions where it is preferable to use paternally inherited polymorphisms to confirm the presence of fetal DNA instead of detecting methylated *RASSF1A*. Several recent studies have reported that aberrant methylation in the promoter region of *RASSF1A* can also be used as potential marker for (early) diagnosis of several types of cancer [34]–[36]. This could mean that there is a potential risk for false positive results in the *mRASSF1A*-PAP assay. Although this risk is considered to be small, given the prevalence of cancer in the reproductive age group, it should be taken into account when including women for NIPD. When there is a history of cancer, this should be reported to the lab which is testing the samples. In these cases, testing of paternally inherited polymorphisms to confirm the presence of fetal DNA is preferable over testing methylated *RASSF1A*.

Although the percentage cffDNA early in gestation differs between individuals, most reports agree that the fetal contribution is around 10% in the first trimester [30], [37], [38]. On average, we isolate 2–3 ng of total cfDNA from maternal plasma, thus expecting around 200–300 pg cffDNA as input for the the mRASSF1A-PAP assay. Our data show that using PAP, we can reproducibly detect amounts much lower than these average expected amounts of fetal DNA, even in the range of a few genome equivalents (30 pg). This demonstrates the extreme sensitivity of PAP. Using serial dilutions, we could even detect amounts in the range of only 1–2 genome equivalents (6–15 pg). However, since only a few copies of the gene of interest are present, these results were less reproducible. We have used this *mRASSF1A*-PAP assay as a control test in fetal sexing. However, it can also be useful in other applications such as noninvasive prenatal testing (NIPT) for fetal trisomies using Next Generation sequencing. Since the assay is universal and sex-independent, it can be applied to all samples and reliably confirms the presence of fetal DNA within a sample.

In conclusion, this study confirmed that methylated *RASSF1A* sequences can be used as informative universal markers for detecting the presence of cffDNA in maternal plasma, irrespective of fetal sex. Moreover, the PAP technique used provides an extremely sensitive method for the detection of fetal sequences in a large pool of maternal plasma DNA early in gestation. Therefore, this assay could be of great value as an addition to current techniques used in noninvasive prenatal diagnostics.
